# Screening and fermentation medium optimization of a strain favorable to Rice–fish Coculture

**DOI:** 10.3389/fmicb.2022.1054797

**Published:** 2022-11-10

**Authors:** Banghua Xia, Haobo Zou, Linyuan Li, Bitao Zhang, Yifang Xiang, Yuning Zou, Zhentao Shen, Shuqun Xue, Ying Han

**Affiliations:** ^1^College of Animal Science and Technology, Northeast Agricultural University, Harbin, China; ^2^China Animal Husbandry Industry Co., Ltd., Beijing, China

**Keywords:** Rice-fish coculture, bacillus licheniformis, probiotic bacteria, one-factor-at-a-time, response surface methodology

## Abstract

Rice–fish coculture (RF) is a small ecosystem in which microorganisms are widely distributed in the fish, water environment, soil, and plants. In order to study the positive effects of microorganisms on common carp and rice in the RF ecosystem, a total of 18 strains with growth-promoting ability were screened from common carp (*Cyprinus carpio*) gut contents, among which three strains had the ability to produce both DDP-IV inhibitors and IAA. The strain with the strongest combined ability, FYN-22, was identified physiologically, biochemically, and by 16S rRNA, and it was initially identified as *Bacillus licheniformis.* As the number of metabolites secreted by the strain under natural conditions is not sufficient for production, the FYN-22 fermentation medium formulation was optimized by means of one-factor-at-a-time (OFAT) experiments and response surface methodology (RSM). The results showed that, under the conditions of a soluble starch concentration of 10.961 g/l, yeast concentration of 2.366 g/l, NH_4_Cl concentration of 1.881 g/l, and FeCl_3_ concentration of 0.850 g/l, the actual measured number of FYN-22 spores in the fermentation broth was 1.913 × 10^9^ CFU/ml, which was 2.575-fold improvement over the pre-optimization value. The optimized fermentation solution was used for the immersion operation of rice seeds, and, after 14 days of incubation in hydroponic boxes, the FYN-22 strain was found to have a highly significant enhancement of 48.31% (*p* < 0.01) on the above-ground part of rice, and different degrees of effect on root length, fresh weight, and dry weight (16.73, 17.80, and 21.97%, respectively; *p* < 0.05). This study may provide new insights into the fermentation process of *Bacillus licheniformis* FYN-22 and its further utilization in RF systems.

## Introduction

Increasing production costs of rice monoculture and concerns about farmers’ food security have prompted farmers to adopt integrated Rice–fish coculture (RF) (Arunrat and Sereenonchai, 2022). RF is being promoted on a large scale by the Chinese government for its ability to save large amounts of water and land resources, promote sustainable aquaculture, boosting rice yields, and reduce the use of chemical fertilizers, while also reducing poverty to a certain extent (Prein, 2002; Frei and Becker, 2005; Luo, 2018; Yuan et al., 2022). As of 2020, RF has a farming area of up to 9,596 km^2^ and fish production of up to 856,900 tons, accounting for 41.41% of the total RF farming area and 29.41% of the total fish production in China, occupying a very important position (FDMARA (Fisheries Department of Ministry of Agriculture Rural Affairs), 2021; Arunrat et al., 2022). Some studies have proven that proper fish farming in RF ecosystems does not reduce the yield of rice, but rather reduces pests and diseases, in addition to controlling weeds (Nico et al., 2002; Vromant et al., 2002), thus reducing the use of herbicides as well as pesticides (Barbara et al., 2018) and even improving the yield and quality of the plants and animals grown (Jinzhao et al., 2020). In addition, compared to conventional rice cultivation or fish farming, rice in RF can take advantage of the nitrogen-containing elements in the excretion of farm animals and the fixation of microorganisms to reduce the input of exogenous nitrogen and accelerate the recycling of nitrogen, thus reducing the use of fertilizers (Jinfei et al., 2016). As an important agent of nutrient cycling in RF, microorganisms play an important role in nutrient cycling, formation, and maintenance of soil fertility and ecological improvement, on one hand (Insam et al., 1996); on the other hand, they colonize the intestinal tract of fish and, thus, colonize the intrinsic intestinal flora forming a micro-ecosystem in which the intestinal microorganisms and the host and the aquatic environment in which they live are mutually regulated and interdependent (Zhang et al., 2014).

The genus *Bacillus* is one of the more studied genera of plant-promoting endophytic bacteria. The main common species of *Bacillus* are *Bacillus cereus*, *Bacillus licheniformis*, *Bacillus amyloliquefaciens*, and *Bacillus subtilis*. It has been found that *Bacillus* spp. are easy to isolate and purify, while producing heat-resistant and resistant bacilli, and the formulation is stable, strongly inhibitory, easy to apply, and transportable, making it an important disease-promoting microorganism (Yeong et al., 2020). *Bacillus licheniformis* is a parthenogenic Gram-positive bacterium that is highly stable and resistant to high temperatures and acidic conditions in the form of endospores. *Bacillus licheniformis* can secrete a variety of digestive enzymes, growth factors, and antibacterial substances, which can effectively promote the degradation of nutrients and assist the body’s energy metabolism, while also promoting the colonization of beneficial bacteria by competing with harmful bacteria for colonization sites, which can play a role in improving intestinal health (Lu et al., 2020). The action of *Bacillus licheniformis* in promoting plant growth includes both direct and indirect mechanisms. The direct growth-promoting effect is mainly through the synthesis of compounds required for plant growth, such as indole acetic acid (IAA), ACC deaminase, cytokinin (CTK), and gibberellins (GAs), or through nitrogen fixation, as well as phosphorus and potassium removal, to increase the content of effective elements such as nitrogen, phosphorus, and potassium that can be directly absorbed in the soil environment (Chunquan et al., 2000; Rana et al., 2011; Rais et al., 2017); the indirect promotion effect is by improving the structure of the soil microbial community (Wang et al., 2019). As a plant growth regulator, IAA can promote plant root growth, increase root length and the number of lateral root growth, and accelerate the uptake and conversion efficiency of soil nutrients during crop growth (Teintze et al., 1981), as well as stimulate plants to secrete ACC deaminase (Kim et al., 2008).

As the level of metabolites secreted by the strain under natural conditions is not sufficient for production, the level of metabolites in the fermentation broth is indirectly enhanced by raising the concentration of *Bacillus licheniformis* spores in the fermentation broth. It is well known that different *Bacillus licheniformis* strains require specific media to achieve maximum conidial production; thus, we optimized a fermentation medium formulation suitable for *Bacillus licheniformis* (FYN-22) by means of mathematical modeling. One-factor-at-a-time (OFAT) experiments represent a classical and effective screening method, which allows for the initial screening of fermentation medium formulations and the determination of the corresponding concentration range (Jawan et al., 2021). Response surface methodology (RSM) is a relatively new statistical method that allows the response value of a system to be visualized as a function of one or more factors and the test results to be represented graphically (Zhilong et al., 2009). RSM uses a multiple quadratic regression equation to fit a functional relationship between factors and response values, and the regression equation can be analyzed to obtain optimal operating parameters that can solve multivariate problems. Both OFAT and RSM have their limitations, and, by using a combination of the two methods, their shortcomings can be compensated for to obtain the desired experimental results (Venkatachalam et al., 2019; Mulatu et al., 2021; Willig et al., 2022).

Therefore, in this study, FYN-22 (*Bacillus licheniformis*) was screened from the intestine of common carp (*Cyprinus carpio*) cultured in RF, and the culture conditions for FYN-22 were optimized using a mathematical model. The effect of the optimized strain was verified *via* a growth promotion test on LJ-31 rice seedlings to evaluate the probiotic effect of the strain in the RF system.

## Materials and methods

### Experimental material

In September 2021, a population of common carp cultured, three healthy 1st -year common carp (150 ± 10 g), was randomly selected from a paddy field in Dawa District, Panjin City, Liaoning Province, and fed only basal feed (mainly consisting of fish meal, soybean meal, vegetable meal, soybean oil, and vitamins and minerals required for common carp growth) during rearing, in a soil environment of white pulpy rice soil (1.03 g/cm^3^ capacity, 61.5% porosity, and the average saturated water content of 0–30 cm volume was 55.6%, and pH 6.52). Common carp were anesthetized with MS-222 anesthetic (250 mg/l). The surface of the common carp was wiped with anhydrous ethanol, and it was then dissected with sterilized scissors and forceps on an ultraclean bench. The whole intestine of the common carp was removed, and the contents were gently squeezed out into a conical flask containing glass beads and 50 ml of sterile water, before shaking at 180 rpm for 30 min at room temperature. This was followed by gradient dilution with 100 μl of 10^−3^, 10^−4^, and 10^−5^ gradients applied to LB solid medium plates; each gradient was repeated three times and incubated at 28°C for 48 h. After 48 h of incubation, strains of different shapes were selected for isolation. All procedures were approved by the local ethics committee and followed by the European Directive 2010/63/EU for animal experiments.

### Determination of IAA activity

Colony isolates from common carp gut contents were inoculated into R_2_A liquid medium containing l-tryptophan and incubated for 4 days at 28°C on a constant shaker shaking at 180 rpm. Then, 500 μl of bacterial suspension was pipetted into a 2 ml glass vial, and 500 μl of Salkowski’s colorimetric solution was added. IAA (500 mg/l) was also added to the Salkowski colorimetric solution as a positive control. The 2 ml glass vials were stored at room temperature and protected from light for 30 min before observing the color change. A red color indicated the ability of the strain to produce IAA in the presence of L-typtophan.

Next, 10 mg of IAA was precisely weighed and dissolved in a small amount of anhydrous ethanol, before adding distilled water to fix the volume to 100 ml, thus configuring a solution of IAA at a concentration of 100 μg/ml as a stock solution. The stock solution was then diluted and configured into a series of standards at concentrations of 0 (blank), 0.5, 1.0, 5.0, 10.0, 15.0, 20.0, and 25.0 μg/ml as working solutions. Then, 2 ml of the above working solutions was added to eight test tubes, before adding two volumes of Salkowski’s colorimetric solution. The solution was kept warm at 40°C for 30 min, and then the absorbance was measured at 530 nm. The IAA standard curve was plotted using OD_530_ as the horizontal coordinate and IAA concentration as the vertical coordinate. The IAA production capacity of the strains was quantified under the same culture conditions as the primary screen. The OD value of the suspension at 530 nm was determined spectrophotometrically; then, the suspension was centrifuged at 10,000 rpm for 10 min, and the supernatant was added to an equal volume of Salkowski’s colorimetric solution and left for 30 min at 40°C, protected from light, to develop the color and determine the OD value at 530 nm. The concentration of IAA per unit volume of fermentation broth was calculated for an OD_530_ value of 1 (an appropriate dilution is required for higher concentrations of bacterial broth) (Chhetri et al., 2022).

### Determination of DDP-IV-inhibitory activity

Colony isolates from the common carp intestinal contents were spread on SKM (2% skim milk) medium plates, and each gradient was repeated three times and incubated at 28°C for 48 h. Screening was carried out using the lactoprotein hydrolysis circle method, and isolates that produced clear circles were picked for purification. The supernatant was removed by centrifugation at 8000 rpm for 15 min, and the slurry was left. The supernatant was washed three times with 0.1 mol/l sterile phosphate-buffered saline (PBS, pH = 6.8) and resuspended in PBS, and the absorbance value was adjusted to 1.0. The supernatant was incubated at a suitable temperature for 24 h, centrifuged at 4°C for 15 min at 8000 rpm, and filtered through a 0.22 pm aqueous membrane. The supernatant was filtered through a 0.22 pm aqueous membrane filter to obtain cell-free excretory supernatants (CFS), which were stored at −80°C.

For strains showing a clear lactoprotein hydrolysis circle, 25 μl of 1.6 mmol/l C_21_H_22_N_4_O_6_ and 25 μl of CFS were added dropwise to a 96-well plate for 15 min at 37°C, followed by 50 μl of 0.01 U/ml DDP-IV for 1 h at 37°C and 100 μl of 1 mol/l The reaction was terminated by the addition of 100 μl of 1 mol/l sodium acetate buffer solution (pH = 4.0), and the absorbance of the reaction solution was measured at 405 nm using an enzyme marker. The DDP-IV inhibition rate was calculated as follows (Hamendra et al., 2012):

(1) $$Rateofinhibition=\left(1-\frac{\Delta X-\Delta P}{\Delta S-\Delta T}\right)\times 100\%$$

where *X* is 25 μl of sample + 25 μl of Gly-Pro-Phy + 50 μl of DDP-IV + 100 μl of sodium acetate buffer solution, *P* is 25 μl of sample + 50 μl of Tris–HCl + 25 μl of Gly-Pro-Phy + 100 μl of sodium acetate buffer solution, *S* is 25 μl of Tris–HCl + 25 μl of Gly-Pro-Phy + 50 μl of DDP-IV + 100 μl of sodium acetate buffer solution, and *T* is 75 μl of Tris–HCl + 25 μl of Gly-Pro-Phy + 100 μl of sodium acetate buffer solution.

### Bacteria resistance testing

Acid resistance test: The strains were inoculated at 2% (*v*/*v*) into LB liquid medium at pH 2.0, 3.0, and 4.0 and incubated at 37°C for 24 h. The number of viable bacteria was determined, and the survival rate of the strains was calculated.

Bile salt tolerance test: The activated strain was diluted in sterile saline at a multiple of 1 ml in a sterile Petri dish. The dishes were then poured with LB solid medium containing 0.5, 1.0, and 1.5% sodium taurocholate, with LB solid medium without sodium taurocholate used as the control group, and incubated at 37°C for 48 h. Colony counting was carried out, and the survival rate of the strains was calculated.

### Identification of strains

16S rRNA identification: A total of 18 different morphological strains with growth-promoting ability were screened from the common carp intestinal contents, and strain FYN-22 with the best growth-promoting activity was selected for 16S rRNA detection. The bacterial genomic DNA extraction kit from Beijing Solabao Biotechnology was used to extract the DNA. The PCR amplification system was a 25 μl system containing: 10× buffer (2.5 μl), Taqase (0.5 μl), primer 27F (0.5 μl), primer 1492R (0.5 μl), DNA template (1 μl), and ddH_2_O (20 μl). The reaction procedure was set to pre-denaturation at 95°C for 5 min, denaturation at 94°C for 50 s, annealing at 56°C for 30 s, extension at 72°C for 1.5 min, 30 cycles of extension at 72°C for 10 min, and storage at 4°C. The PCR amplification products were sent to RuiBiotech for sequencing. The 16S rRNA sequencing results of the strains were compared using the NCBI database, and a phylogenetic tree was constructed (Yao et al., 2022).

Physiological and biochemical identification: The conserved strains were triplicated on solid LB medium plates. Single colonies were isolated, and their morphology was described. The strains were subjected to Gram staining and physiological and biochemical identification according to Bergey’s Manual of Determinative Bacteriology.

## Media optimization

### One-factor-at-a-time (OFAT) experiments

Selection of the best carbon source: Bean sprout juice medium (bean sprout juice 100 ml, sucrose 10 g, (NH_4_)_2_SO_4_ 2 g, NaCl 0.6 g, distilled water fixed to 1,000 ml, pH adjusted to 7, autoclaved at 115°C for 30 min) was selected as the base medium, and 1% of C_6_H_12_O_6_, C_5_H_10_O_5_, C_12_H_22_O_11_, β-C_12_H_22_O_11_ − H_2_O, soluble starch, and corn starch were added to replace the original carbon source. The seed solution was inoculated at 0.5% inoculum and incubated at 180 rpm for 15 h at 37°C. The optimal concentration of carbon source in the medium was determined by changing the optimal carbon source concentration and incubating again.

Selection of the best organic nitrogen source: Peptone meat, peptone germ, peptone soya, peptone casein, casein tryptone, yeast, and soybean meal (0.2%) was added to the fermentation medium as a nitrogen source and carbon source, with the above fermentation conditions.

Selection of the best inorganic nitrogen source: CH_4_N_2_O, NH_4_Cl, NH_4_NO_3_, NaNO_3_, (NH_4_)_2_C_2_O_4_, and NH_4_H_2_PO_4_ (0.2%) were added to the fermentation medium as inorganic nitrogen sources, along with the corresponding best carbon source and best organic nitrogen source, under the same fermentation conditions as above.

Optimal inorganic salt screening: FeCl_3_, MgSO_4_, CuSO_4_, ZnSO_4_, CaCO_3_, FeSO_4_, and MnCl_2_ (0.06%) were added to the fermentation medium as inorganic salts, along with the corresponding optimal carbon and nitrogen sources, under the same fermentation conditions as above.

### Box–Behnken design and response surface analysis

On the basis of the OFAT test, the effect of variations in fermentation medium formulation on the spore concentration of *Bacillus licheniformis* FYN-22 fermentation broth was explored using a Box–Behnken design (BBD) and RSM methods with four factors (carbon source, organic nitrogen source, inorganic nitrogen source, and inorganic salt) and three levels (−1, 0, and 1) (Table 1). The experimental design and statistical analysis were carried out using Design-Expert software, with a total of 29 sets of experiments, including five centroids. A quadratic polynomial was used to relate the relationship between the independent variables and the corresponding values (Equation (2)):


(2) $$Y=\beta_{0}+\sum \beta_{i}x_{i}+\sum \beta_{\mathrm{ij}}x_{i}x_{j}+\sum \beta_{\mathrm{ii}}x_{i}^{2}$$

where *Y* is the predicted response, *x*_i_ and *x*_j_ are independent factors, *β*_0_ is the model intercept, *β*_i_ is the linear coefficient, *β*_ii_ is the quadratic coefficient, and *β*_ij_ is the interaction coefficient.

**Table 1:** Coded and actual values of the variables for the four-factor Box–Behnken experimental design.

Variables	Symbol	Coded and Actual Values		
		−1	0	1
Soluble starch (× 10^−2^ g/ml)	*A*	1.2	1.0	1.4
Yeast (× 10^−3^ g/ml)	*B*	2.0	2.5	3.0
NH_4_Cl (× 10^−3^ g/ml)	*C*	1.0	1.5	2.0
FeCl_3_ (× 10^−3^ g/ml)	*D*	0.7	0.8	0.9

### Effect of FYN-22 strain On The growth of Rice seedlings

Rice LJ31 was used as the test rice. Rice seeds of uniform size and full morphology were selected from each treatment group and disinfected by submerging the seeds in 70% ethanol for 15 min, before rinsing three times with sterile water to remove the ethanol residue. Disinfected rice seeds were placed in a 100 ml conical flask with 50 ml of the corresponding soaking solution and placed in an incubator for 2 days at 28 ± 0.5°C. After 2 days of germination, the uniformly germinated seeds were placed in a hydroponic box and placed in a plant light incubator for 14 days at 28°C at room temperature. Two groups of treatments were set up: sterile water immersion followed by normothermic hydroponics (control group) and optimized *Bacillus licheniformis* FYN-22 (spore concentration of 1.913 × 10^9^ CFU/ml) immersion followed by normothermic hydroponics (treatment group). The plant light incubator conditions were as follows: day/night light duration 12 h/12 h, light intensity 12,000 lx, and humidity 60%.

### Data analysis and software

Data were analyzed using Design-Expert 12.0.3.0 and SPSS 13.0, with MEGA 7.0.14 used for plotting phylogenetic trees and GraphPad Prism 8.0.1 software used for graphing. Statistical analysis was performed using SPSS statistical software. Experiments were repeated a minimum three times, and all data are expressed as the mean standard error (SD).

## Results

### Screening and identification of bacteria and evaluation of growth-promoting activity

**Figure 1:**
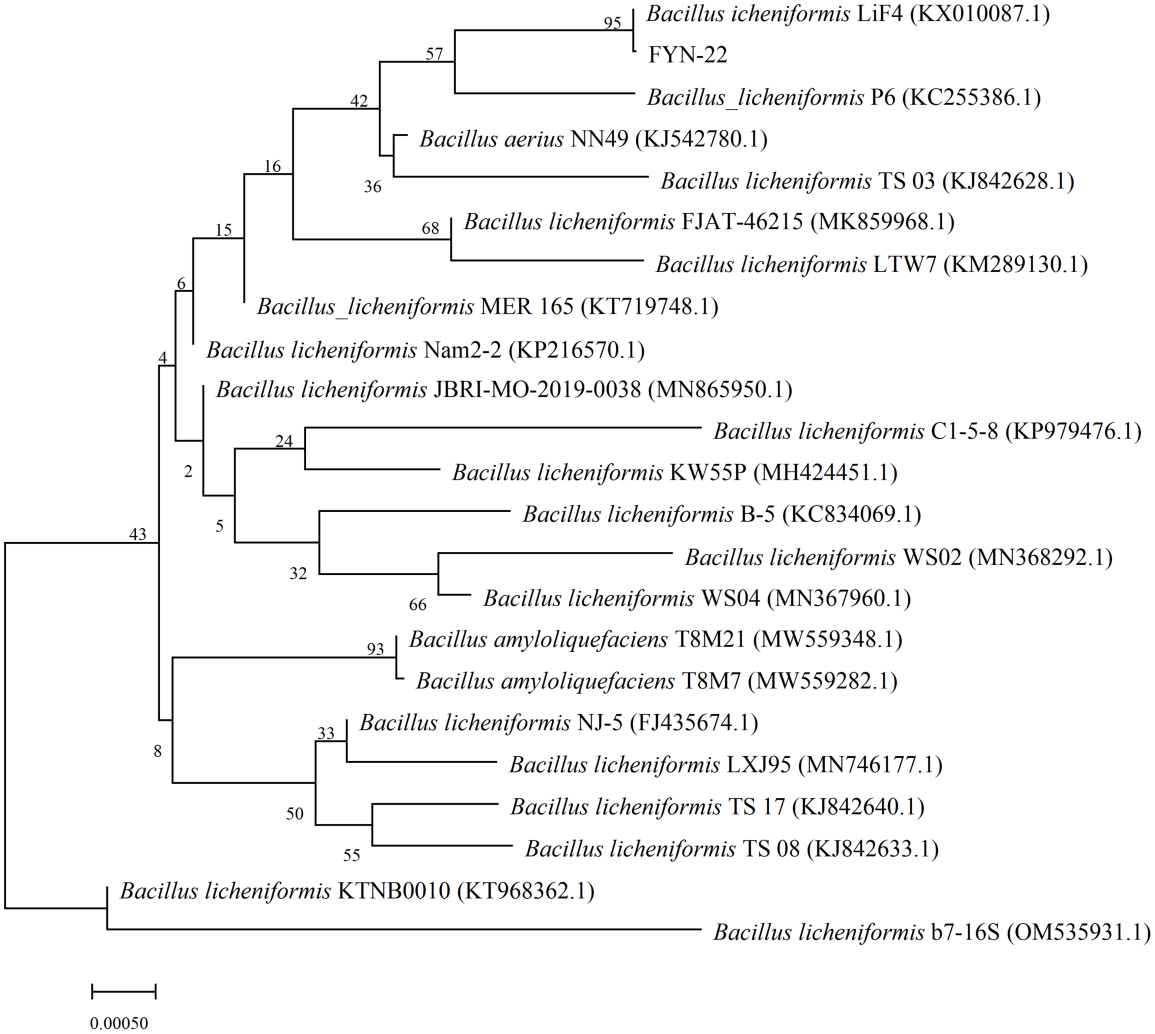
Phylogenetic tree was constructed based on the 16S rRNA sequence of the FYN-22 strain. The numbers at the nodes indicate the percentages of bootstrap sampling derived from 1,000 replications. GenBank accession numbers are given in parentheses. 0.00050 in the legend is the distance scale.

As shown in Table 2, a total of 18 different morphological strains with growth-promoting ability were screened from the common carp intestinal contents. Twelve of these strains had the ability to produce DDP-IV inhibitors (FLN-1, FLN-4, FLN-25, FLN-26, FYN-1, FYN-2, FYN-4, FYN-8, FYN-9, FYN-11, FYN-14, and FYN-22). Among them, strain FYN-14 had the highest DDP-IV inhibition rate of 62.40% (Supplementary Figure S1). Ten strains had the ability to produce IAA (FLN-4, FLN-37, FYN-3, FYN-8, FYN-10, FYN-11, FYN-16, FYN-21, FYN-22, and FYN-38), with FLN-37 having the strongest ability to produce IAA growth hormone at 120.49 μg/ml (Supplementary Figure S2). Three strains (FLN-4, FYN-8, and FYN-22) had the ability to both inhibit DDP-IV and produce IAA, with the best combined effect being seen for FYN-22 (58.72% DDP-IV inhibition and 118.55 μg/ml IAA content). Therefore, the 16S rRNA of strain FYN-22 was sequenced, and a phylogenetic tree was constructed (Figure 1), from which it can be seen that strain FYN-22 was in the same smallest clade as *Bacillus licheniformis* (KX010087.1), with a close evolutionary distance. According to the physiological and biochemical indices (Table 3), strain FYN-22 (SUB12101904 FYN-22 OP535852) was tentatively identified as *Bacillus licheniformis*(*Bacillus Cohn*).

**Table 2:** Detection of FYN-22 strain with DDP-IV-inhibitory activity and IAA activity. ‘+’ denotes presence of the ability; ‘−’ denotes absence of the ability.

Strain name	DDP-IV Inhibitor activity	DDP-IV Inhibitor Inhibition rates (%)	IAA Activity	IAA Content (μg/mL)
FLN-1	+	44.38	−	−
FLN-4	+	29.76	+	75.44
FLN-25	+	17.54	−	−
FLN-26	+	26.30	−	−
FLN-37	−	−	+	120.49
FYN-1	+	13.87	−	−
FYN-2	+	35.43	−	−
FYN-3	−	−	+	56.86
FYN-4	+	60.59	−	−
FYN-8	+	27.68	+	28.77
FYN-9	+	52.45	−	−
FYN-10	−	−	+	43.28
FYN-11	+	33.42	+	87.34
FYN-14	+	62.40	−	−
FYN-16	−	−	+	37.69
FYN-21	−	−	+	105.49
FYN-22	+	58.73	+	118.55
FYN-38	−	−	+	13.80

**Table 3:** Detection of physiological and biochemical parameters of the FYN-22 strain. ‘+’ denotes presence of the ability; ‘−’ denotes absence of the ability.

Examining Item	Results	Examining Item	Results
Gram stain test	Positive	Arabinose	+
Contact enzyme test	+	Xylose	+
Oxidase test	+	Mannose	−
Voges–Proskauer test	+	Glucose production test	−
Methyl red test	+	Hydrolysis of starch	+
Reduction of nitrate	+	Citrate	+
Glucose	+	Growth at 50°C	+

### Resistance test for FYN-22 strain

Bacteria perform their probiotic functions by colonizing the gastrointestinal tract; hence, the strains must be tolerant to the acidity of the gastric juice and the bile salts in the intestine in order to perform their probiotic functions. As can be seen from Table 4, the effect on FYN-22 spore concentration was lower at pH 3.0 and 4.0, and a significant decrease in FYN-22 spore concentration occurred at pH 2.0, while there was no significant decrease in FYN-22 spore concentration when the bile salt concentration was increased. This result indicates that FYN-22 strain has a high tolerance to pH and bile salts, and it can maintain a relatively high activity upon reaching the intestine after passing through the gastric juice.

**Table 4:** Acid and bile salt tolerance test for FYN-22 strain.

FYN-22 Initial spore concentration (× 10^8^ CFU/ml)	pH	FYN-22 spore concentration (× 10^8^ CFU/ml)	Survival rate (%)	FYN-22 Initial spore concentration (× 10^8^ CFU/ml)	Bile salt concentration (%)	FYN-22 spore concentration (× 10^8^ CFU/ml)	Survival rate (%)
3.78	2.0	3.25	85.98	3.62	0.5	3.39	93.65
	3.0	3.52	93.12		1.0	3.27	90.33
	4.0	3.64	96.30		1.5	3.16	87.29

### Effect of modified medium components on the concentration of FYN-22 spores using OFAT

**Figure 2:**
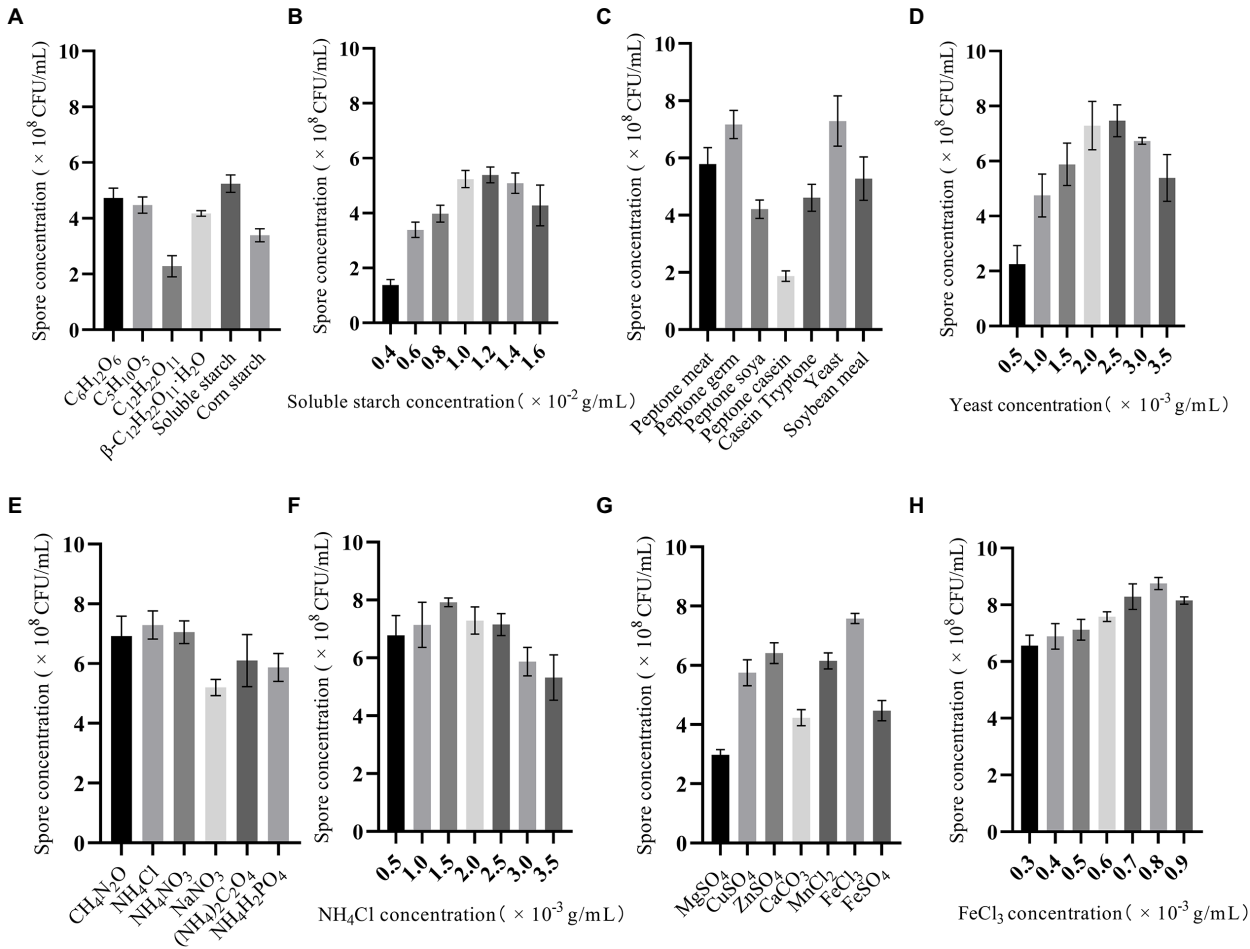
Effect of OFAT optimized carbon source composition **(A)**, soluble starch concentration **(B)**, organic nitrogen source composition **(C)**, yeast concentration **(D)**, inorganic nitrogen source composition **(E)**, NH_4_Cl concentration **(F)**, inorganic salt composition **(G)**, and FeCl_3_ concentration **(H)** on the concentration of FYN-22 spores. Error bars indicate standard error (*n* = 3).

During the optimization of the FYN-22 fermentation medium formulation, OFAT was first used to screen the types and initial concentration ranges of carbon, inorganic nitrogen, organic nitrogen, and inorganic salts, before the medium was further optimized with RSM. To the base fermentation medium, 1% of C_6_H_12_O_6_, C_5_H_10_O_5_, C_12_H_22_O_11_, β-C_12_H_22_O_11_–H_2_O, soluble starch, and corn starch were added as carbon sources. Figure 2A shows that different carbon sources had a relatively significant effect on FYN-22 spore concentration, with the highest spore concentration of 5.24 × 10^8^ CFU/ml for FYN-22 when soluble starch was added as a carbon source.

The effect of the content of soluble starch when used as a carbon source on the spore concentration of FYN-22 was investigated by varying the amount of soluble starch added, and the spore concentration of FYN-22 reached a maximum value of 5.39 × 10^8^ CFU/ml when soluble starch was added at 1.2 × 10^−2^ g/ml (Figure 2B). The spore concentration of FYN-22 was higher than the other six organic nitrogen sources when yeast was used as the organic nitrogen source (Figure 2C), reaching a maximum of 7.47 × 10^8^ CFU/ml when yeast was added at 2.5 × 10^−3^ g/ml (Figure 2D). Compared to organic nitrogen sources, inorganic nitrogen sources are relatively homogeneous in composition and more stable in quality, making them more readily available for rapid use by microorganisms in fermentation media. CH_4_N_2_O, although organic, is considered an inorganic form of nitrogen in this experiment as it provides ammonium nitrogen when used as a nitrogen source for the media. As shown in Figures 2E,F, the spore concentration of FYN-22 reached a maximum of 7.92 × 10^8^ CFU/ml when NH_4_Cl was used as the inorganic nitrogen source at a concentration of 1.5 × 10^−3^ g/ml. From Figure 2G, it can be seen that different types of inorganic salts had a significant effect on the spore concentration of FYN-22. When FeCl_3_ was selected as the inorganic salt to be added to the fermentation medium, the spore concentration of FYN-22 was significantly enhanced compared to other inorganic salts. The spore concentration of FYN-22 reached a maximum value of 8.75 × 10^8^ CFU/ml when the FeCl_3_ concentration was 0.8 × 10^−3^ g/ml (Figure 2H). From the above results, it was shown that the spore concentration of FYN-22 could be influenced to some extent by changing the type and content of the different components of the fermentation medium.

### Effect of modified medium components on the concentration of FYN-22 spores using RSM

According to the OFAT experiment, the results of the formulation test for the fermentation culture of FYN-22 were obtained using the Design-Expert software with the addition of soluble starch (A), Yeast (B), NH_4_Cl (C), and FeCl_3_ (D) as test factors and the concentration of FYN-22 spores as the response variable (Table 5). Multiple regressions were fitted to the response values to obtain a quadratic regression model of FYN-22 spore concentration (Y) as a function of the four coded independent variables (Equation (3)), and analysis of variance (ANOVA) was performed on the regression equations. As can be seen from Table 6, the quadratic term model chosen for this test was highly significant (*p* < 0.01); the lack of fit (*p* = 0.0731) was not significant (>0.05), indicating that the test data were highly consistent with the model. The coefficient of variation (CV) reflects the confidence and accuracy of the test validation, and the CV obtained in this test was 2.72% (<10%), indicating that the equation had a high degree of confidence and accuracy (Yu et al., 2009). The coefficient of determination *R*^2^ = 0.9216 and the corrected coefficient of determination Adj-*R*^2^ = 0.8433 indicate that this equation was a good fit, and the model could successfully be used for prediction. In the regression model, the primary terms *A*, *B*, *C*, and *D* had a large effect on the response values and all reached a highly significant level (*p* < 0.01); the interaction term *AB* reached a significant level (*p* < 0.05), and the secondary terms *C*^2^ and *D*^2^ also reached a significant level (*p* < 0.05), indicating that the effect of each factor on the concentration of FYN-22 spores was not a simple linear relationship. The *F*-value reflects the importance of each factor with respect to the response variable, with a larger *F*-value indicating a greater influence. From the magnitude of the *F*-values in Table 6, it can be judged that the order of influence of the four factors on FYN-22 spore concentration within the selected level range was soluble starch (15.25) > NH_4_Cl (14.76) > yeast (13.81) > FeCl_3_ (11.57).


(3) $$\begin{array}{c} Y=1.84-0.0517A-0.0492B+0.0505C+\\ 0.0450D+0.0650AB–0.0275AC–0.0275AD+\\ 0.0001BC–0.0075BD+0.0100CD–0.0997A^{2}–\\ 0.01610B^{2}-0.0460C^{2}–0.0698D^{2} \end{array}$$

Here, *Y* is the spore concentration of FYN-22, *A* is the coded value of soluble starch, *B* is the coded value of yeast, *C* is the coded value of NH_4_Cl, and D is the coded value of FeCl_3_.

**Table 5:** Response surface methodology design and results.

Exp.Run	Factors				Spore concentration (× 10^9^ CFU/ml)
	Soluble starch (× 10^−2^ g/ml)	Yeast (× 10^−3^ g/ml)	NH_4_Cl (× 10^−3^ g/ml)	FeCl_3_ (× 10^−3^ g/ml)	
1	1.2	2.5	1.5	0.8	1.81
2	1.2	2.5	1.5	0.8	1.84
3	1.2	2.5	1.5	0.8	1.87
4	1.2	2.5	1.5	0.8	1.86
5	1.2	2.5	1.5	0.8	1.83
6	1.2	3.0	1.5	0.9	1.64
7	1.2	2.0	1.5	0.9	1.75
8	1.2	3.0	1.5	0.7	1.5
9	1.2	2.0	1.5	0.7	1.58
10	1.4	2.5	2.0	0.8	1.67
11	1.0	2.5	2.0	0.8	1.88
12	1.4	2.5	1.0	0.8	1.58
13	1.0	2.5	1.0	0.8	1.68
14	1.2	3.0	2.0	0.8	1.63
15	1.2	2.0	2.0	0.8	1.74
16	1.2	3.0	1.0	0.8	1.52
17	1.2	2.0	1.0	0.8	1.63
18	1.4	2.5	1.5	0.9	1.62
19	1.0	2.5	1.5	0.9	1.71
20	1.4	2.5	1.5	0.7	1.68
21	1.0	2.5	1.5	0.7	1.66
22	1.2	2.5	2.0	0.9	1.82
23	1.2	2.5	1.0	0.9	1.75
24	1.2	2.5	2.0	0.7	1.68
25	1.2	2.5	1.0	0.7	1.65
26	1.4	3.0	1.5	0.8	1.54
27	1.0	3.0	1.5	0.8	1.53
28	1.4	2.0	1.5	0.8	1.5
29	1.0	2.0	1.5	0.8	1.75

**Table 6:** ANOVA analysis for response surface quadratic model.

Source	Sum of squares	DF	Mean square	*F* value	Prob > F	
Model	0.3459	14	0.0247	11.76	<0.0001	Significant
A (soluble starch)	0.0320	1	0.0320	15.25	0.0016	Significant
B (yeast)	0.0290	1	0.0290	13.81	0.0023	Significant
C (NH_4_Cl)	0.0310	1	0.0310	14.76	0.0018	Significant
D (FeCl_3_)	0.0243	1	0.0243	11.57	0.0043	Significant
AB	0.0169	1	0.0169	8.05	0.0132	Significant
AC	0.0030	1	0.0030	1.44	0.2500	
AD	0.0030	1	0.0030	1.44	0.2500	
BC	0.0001	1	0.0001	0.0001	1.0000	
BD	0.0002	1	0.0002	0.1071	0.7483	
CD	0.0004	1	0.0004	0.1904	0.6692	
A^2^	0.0645	1	0.0645	30.73	<0.0001	
B^2^	0.1681	1	0.1681	80.05	<0.0001	
C^2^	0.0137	1	0.0137	6.53	0.0228	Significant
D^2^	0.0316	1	0.0316	15.02	0.0017	Significant
Residual	0.0294	14	0.0021			
Lack of fit	0.0271	10	0.0027	4.76	0.0731	Not significant
Pure error	0.0023	4	0.0006			
Corr total	0.3753	28				

**Figure 3:**
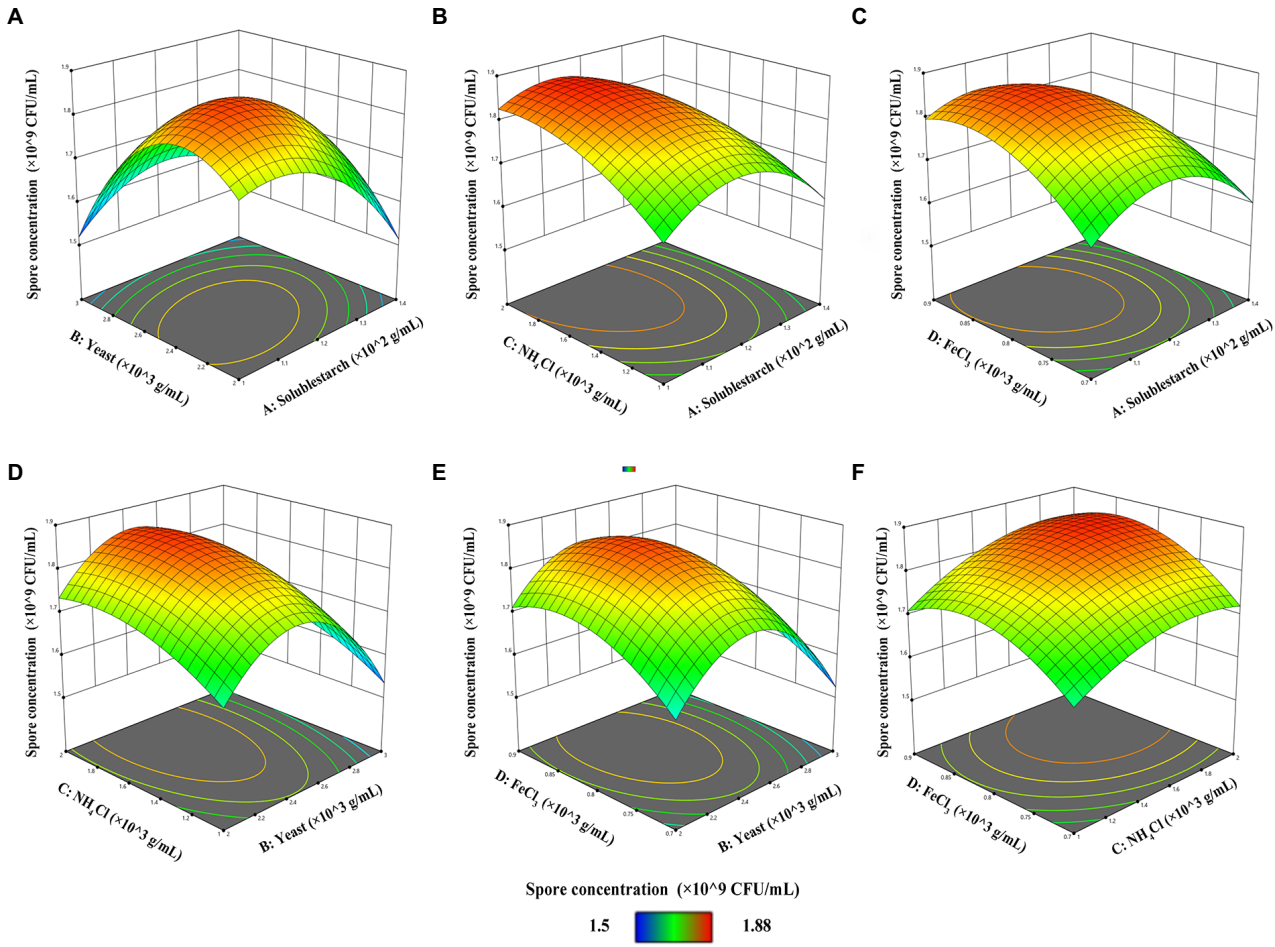
Three-dimensional contour plots of RSM for combined effects of **(A)** soluble starch and yeast, **(B)** soluble starch and NH_4_Cl, **(C)** soluble starch and FeCl_3_, **(D)** yeast and NH_4_Cl, **(E)** yeast and NH_4_Cl, and **(F)** yeast and FeCl_3_ on FYN-22 spore concentration (*n* = 3).

The response surface 3D plot is a graphical representation of the regression equation from which the optimal parameters, the effect of the interaction between the variables on the response value, and the maximum response value can be determined visually and quickly. The contour line is the projection of the response surface in the horizontal direction. An elliptical shape of the contour line indicates a significant interaction between the two factors, while a circular shape indicates an insignificant interaction between the two factors (Kun et al., 2013). Figure 3A demonstrates the effect of the interaction of soluble starch and yeast on the spore concentration of FYN-22 strain when the fixed NH_4_Cl and FeCl_3_ levels were 1.5 × 10^−3^ g/ml and 0.8 × 10^−3^ g/ml, respectively, and the soluble starch concentration increased from 1.0 × 10^−2^ g/ml to 1.4 × 10^−2^ g/ml and that of yeast increased from 2.0 × 10^−3^ g/ml to 3.0 × 10^−3^ g/ml. The spore concentrations of FYN-22 in the fermentation broth all showed a trend of increasing and then decreasing. The highest point of the surface was the maximum point of spore concentration (1.8559 × 10^9^ CFU/ml), the maximum value obtained from the response surface fell in the smallest ellipse in the contour plot, and the contour plot was elliptical, indicating that the interaction between soluble starch and yeast was more significant. Figure 3B illustrates the interaction of soluble starch and NH_4_Cl. According to the contours, it is clear that there was no significant effect of the two factors on FYN-22 spore concentration, whereby the levels of soluble starch (up to 1.13006 × 10^−2^ g/ml) and NH_4_Cl (up to 1.82873 × 10^−3^ g/ml) led to an increase in the concentration of FYN-22 spores. When the levels of soluble starch and NH_4_Cl exceeded 1.13006 × 10^−2^ g/ml and 1.82873 × 10^−3^ g/ml, respectively, there was a greater negative impact on FYN-22 spore concentrations. As shown in Figure 3C, the interaction between soluble starch and FeCl_3_ was not significant. With increasing concentrations of soluble starch and FeCl_3_, the concentration of FYN-22 spores showed a trend of first increasing and then decreasing. When the soluble starch concentration was maintained at 1.1376 × 10^−2^ g/ml, and the FeCl_3_ concentration was maintained at 0.8384 × 10^−3^ g/ml, the FYN-22 spore concentration showed a high level (1.8587 × 10^9^ CFU/ml). As can be seen in Figure 3D, yeast showed a trend of increasing and then decreasing when the concentration of NH_4_Cl was certain, and the slope of the response surface was steep, indicating that yeast had a greater effect on the concentration of FYN-22 spores. When yeast concentration reached 2.4238 × 10^−3^ g/ml, the concentration of NH_4_Cl (1.7762 × 10^−3^ g/ml) had a better promotion effect on FYN-22 spore concentration (1.8598 × 10^9^ CFU/ml); however, at higher levels, both NH_4_Cl and yeast had a negative effect on FYN-22 spore concentration. As can be seen in Figure 3E, the interaction between yeast and FeCl_3_ had no significant effect on FYN-22 spore concentrations. When FeCl_3_ was maintained at a certain concentration, the increase in yeast concentration influenced the change in FYN-22 spore concentration to a greater extent. At a yeast concentration of 2.4198 × 10^−3^ g/ml, the FYN-22 spore concentration reached a high level of 1.8534 × 10^9^ CFU/ml, with a steep slope of the response surface and an overall trend of increasing and then decreasing. As can be seen in Figure 3F, when the concentrations of soluble starch and yeast were fixed at 1.5 × 10^−2^ g/ml and 0.8 × 10^−3^ g/ml, respectively, the NH_4_Cl concentration increased from 1.0 × 10^−3^ g/ml to 2.0 × 10^−3^ g/ml, and that of FeCl_3_ increased from 0.7 × 10^−3^ g/ml to 0.9 × 10^−3^ g/ml. The spore concentration of FYN-22 in the fermentation broth showed a trend of first increasing and then decreasing, with a relatively gentle slope of the surface, at which point the highest point of the surface was the maximum point of spore concentration (1.8653 × 10^9^ CFU/ml).

In summary, the magnitude of the interaction between the factors was in the order of soluble starch and yeast > soluble starch and NH_4_Cl = soluble starch and FeCl_3_ > CNH_4_Cl and FeCl_3_ > yeast and FeCl_3_ > yeast and NH_4_Cl. These results indicate that the content of FYN-22 spore concentration increased rapidly with increasing soluble starch content.

The software was used to solve the regression equation to predict the optimal formulation of *Bacillus licheniformis* FYN-22 fermentation medium within the tested factor levels of soluble starch 10.9605 g/l, yeast 2.3657 g/l, NH_4_Cl 1.8808 g/l, and FeCl_3_ 0.8495 g/l, and an optimized concentration of 1.8925 × 10^9^ CFU/ml of FYN-22 spores was predicted in the fermentation broth. Considering practical feasibility, the medium formulation was amended to soluble starch 10.961 g/l, yeast 2.366 g/l, NH_4_Cl 1.881 g/l, and FeCl_3_ 0.850 g/l. Under these conditions, the actual measured FYN-22 spore concentration in the fermentation broth was 1.913 × 10^9^ CFU/ml, which was 101.083% of the theoretical value (this validation test was repeated three times), and the predicted and measured values were basically the same, indicating that the regression equation could reflect the influence of various factors on the FYN-22 spore concentration in the fermentation broth in a more realistic way.

### Effect of FYN-22 strain on the growth of rice seedlings

**Figure 4:**
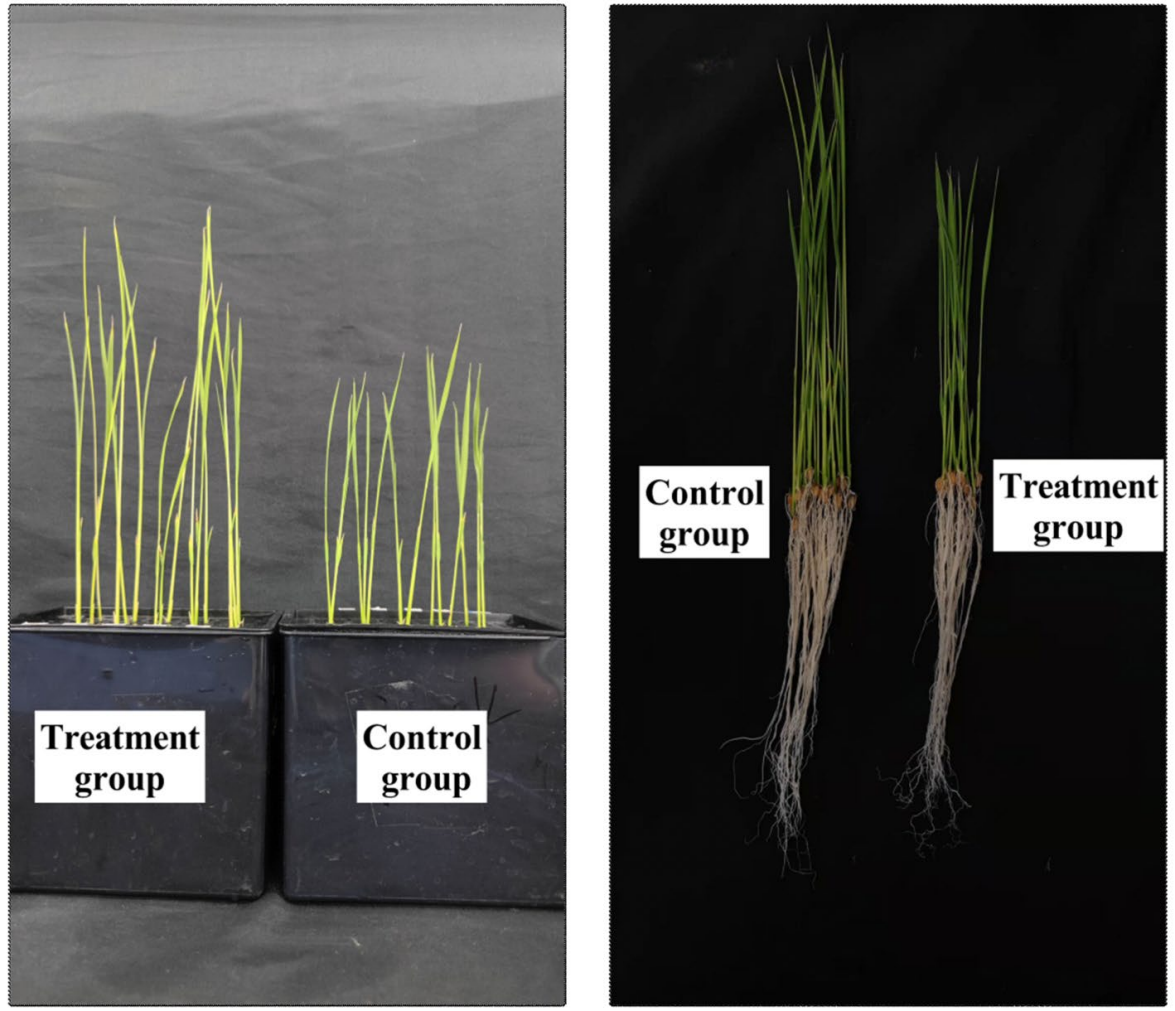
Effect of FYN-22 strain on the growth of rice seedlings.

Rice is mainly grown by transplanting seedlings; hence, the length of the seedling and the morphological structure of the root system play very important roles in the colonization of the rice plant after transplanting, as well as in the later growth of the rice plant. Under hydroponic box culture conditions, the FYN-22 strain had a more pronounced growth-promoting effect on rice (Figure 4). As can be seen from the Table 7, the rice shoot lengths exhibited a highly significant increase (*p* < 0.01), with a 48.31% increase in the treatment group compared to the control. FYN-22 also affected the root length, fresh weight, and dry weight of rice to varying degrees, raising them by 16.73%, 17.80, and 21.97%, respectively (*p* < 0.05).

**Table 7:** Effect of FYN-22 strain on the growth of rice seedlings (*n* = 20).

Groups	Root length (mm)	Shoot length (mm)	Fresh weight (mg)	Dry weight (mg)
Control	9.92 ± 0.49	10.08 ± 0.40	127.33 ± 36.05	20.76 ± 3.41
Treatment	11.58 ± 0.47	14.95 ± 1.29	150.83 ± 36.35	25.32 ± 2.55

## Discussion

Spores are the propagules produced by microorganisms and are an important intermediate in the production of microbial fermentation. The quality and quantity of spores have a significant impact on fermentation yield and biological activity, which are influenced by a combination of factors such as nutrients and the environment. Spore concentration is an important indicator in the determination of biocontrol formulations. Optimization of the culture medium and fermentation conditions to increase spore concentration can provide a guarantee for the industrial production and application of biocontrol formulations. The fermentation conditions during microbial fermentation have a decisive influence on the growth of microorganisms and the production of microbial secondary metabolites. Suitable carbon, nitrogen, and inorganic salts can promote the growth of strains and the production of secondary metabolites (Wen et al., 2020).

The carbon source is one of the most important components of the culture medium, responsible for providing energy for the cells, forming the carbon shelf, and synthesizing metabolites. Common carbon source substances mainly include carbohydrates, lipids, and organic acids. In this study, different carbon source substances were optimized using OFAT experiments, and it was found that soluble starch as a carbon source increased the spore concentration in the fermentation broth of the FYN-22 strain (Figure 2A). Soluble starch is a phytose carbon source that is degraded to glucose during bacterial fermentation, which is then taken up and converted by the cells. Glucose is the preferred carbon source for bacteria, but excess glucose can also inhibit bacterial growth and metabolic metabolism (Hans et al., 2003; Chen et al., 2019). The slow degradation of soluble starch to glucose ensures that there is no significant accumulation of glucose in the medium, thus avoiding the negative effects caused by excess glucose. Yaseen et al. (2017), on the other hand, found that different carbon and nitrogen sources affected the expression of the panglosin promoter, and that the activity of the panglosin promoter could be increased when urea or a mixture of urea and ammonium salts was used as the nitrogen source and when mannitol was used as the carbon source, thus increasing its production (Yaseen et al., 2017).

Nitrogen-derived substances are the main building blocks of proteins and nucleic acids in microbial cells, having an important influence on cell growth and, in turn, on the relevant biochemical reactions in the cell. Due to the complex composition of nitrogen sources, the nutrients contained in different nitrogen source types vary greatly, and the effects of various nitrogen sources on the growth and yield of the bacteria vary significantly. When many kinds of nitrogen source substances are present in the environment at the same time, ammonium salts and glutamine, which are easily absorbed and utilized, can be preferentially utilized (Marzluf, 1997; Hajjaj et al., 2001; Wong et al., 2008). In this study, different nitrogen source substances were optimized using OFAT experiments, and it was found that organic (yeast) and inorganic (NH_4_Cl) nitrogen sources increased the bacterial concentration in the fermentation broth of strain FTN-22(Figures 2C,E). Nitrogen sources are extremely important to *Bacillus licheniformis* (Kim et al., 2008), and yeast contains a wide variety of proteins, inorganic salts, vitamins, and growth-promoting factors that are more nutritious and promote greater protease production than other nitrogen sources (Zhilong et al., 2009; Wang et al., 2019). NH_4_Cl as a secondary nitrogen source can improve the growth rate and enzyme yield of the strain, and the combination of organic and inorganic nitrogen sources can provide nutrients to the strain more fully (Rey et al., 2004).

As a physiologically active substance, inorganic salts regulate the growth of microorganisms and enzyme production and are an indispensable part of the composition of fermentation media. Studies have shown that Na^+^ and K^+^ play a regulatory role in cellular osmotic pressure; Mn^2+^ is an important enzyme activator; Ca^2+^ promotes the production of budding spores in *Bacillus* spp.; Cu^2+^ only promotes the growth of the strain at low concentrations, and, when the concentration increases, it inhibits the growth of the bacterium, while Zn^2+^ only inhibits the growth and reproduction of the strain without promoting it; phosphate can adjust the pH of the fermentation broth and maintain a relatively stable pH of the fermentation broth; Fe^3+^ can be used by iron-containing cells (Pham et al., 2021; Zhang et al., 2021; Kruppa and Czermak, 2022). In this study, different inorganic salts were optimized using OFAT experiments, and it was found that using FeCl_3_ as the source of inorganic salt increased the spore concentration in the fermentation broth of the FYN-22 strain. Iron ions are utilized early in the growth of the bacterium and are re-released into the medium after spore formation; Kolodziej and Slepecky (1964), demonstrated that the addition of iron ions, although not essential for spore formation, increased spore formation (Kolodziej and Slepecky, 1964). Interestingly, Fe^3+^ was more effective than Fe^2+^ in promoting the growth of the FTN-22 strain than the FTN-22 strain (Figure 2G), mainly because Fe^2+^ tends to form chelators with the lipopeptides synthesized by the bacterium, thus inhibiting its promoting effect, whereas Fe^3+^ is not affected by this. The bacterium can form and utilize Fe-containing cells, which mainly take up Fe^3+^; during the subsequent uptake and transport of Fe-containing cells, Fe^3+^ is reduced to Fe^2+^ and utilized by the bacterium.

RSM is a combination of mathematical and statistical methods to seek the best conditions in a multifactor system, to model and analyze response problems influenced by multiple variables, to shorten the optimization time, and to improve the credibility of the application; the method has a low number of experiments, short cycle time, and high accuracy (Tamilarasan et al., 2022). For traditional mathematical and statistical methods, OFAT experiments can only consider the influence of a single factor and cannot determine whether there is an interaction between the factors. Although orthogonal tests can determine the interaction between factors, they require a large number of tests, need explicit functional expressions, and can only deal with discrete-level values. RSM largely compensates for the shortcomings of these two traditional mathematical and statistical methods by not only studying the interaction between several factors, but also predicting the response values outside the region, using a smaller number of trials to obtain highly precise regression equations and, thus, obtaining optimal combinations between multiple experimental variables (Walbi et al., 2022). Currently, RSM is used in food science, medicine, the chemical industry, and engineering. In this study, the optimal range of *Bacillus licheniformis* FYN-22 strains was screened using OFAT experiments, and the optimal fermentation recipe was derived using RSM.

Most of the physiological activities of plants are regulated by one or more plant hormones, mainly IAA, CTK, Gas, abscisic acid (ABA), ethylene, salicylic acid, jasmonic acid, and brassinolide; growth hormones represent the most indispensable part of plants. They have been reported not only in plants, but also in bacteria and fungi (Spaepen and Vanderleyden, 2011; Duca and Glick, 2020). IAA is a growth hormone that acts on the whole process of plant growth and development. IAA affects plant cell division, elongation, differentiation, seed germination, root development, and the process of nutritional growth (Phillips et al., 2011). In dicotyledonous plants, IAA promotes the production of lateral roots; in monocotyledonous plants, it promotes the formation of adventitious roots. The response of IAA to the plant is influenced by light, gravity, and the flowering and fruiting stages of the plant, and it is important in terms of plant photosynthesis, pigment formation, synthesis of various metabolites, and stress resistance (Woodward et al., 2010). Several genera have been reported in the literature to have the ability to secrete IAA (Patten and Glick, 1996). Since 1978 (Tien et al., 1979), when bacteria were first discovered to be able to produce IAA, a large number of IAA-producing strains have been discovered over the past 40 years, with *Pseudomonas* spp. (Ying et al., 2012) and *Bacillus* spp. (Shim et al., 2015) dominating the list. The growth-promoting effect of growth-promoting bacteria applied to substrates on crop seedlings has been demonstrated in seedlings of pepper (Yingnan et al., 2019), cabbage (Henry et al., 2015), and tomato (Cochard et al., 2022). The mechanism of action of probiotic bacteria on crops is firstly manifested in the effect on the crop root system, the morphological structure of which affects the ability of the root system to obtain water and nutrient resources from the soil and furthermore affects the growth of the above-ground parts of the crop (Venkatachalam et al., 2019; Mulatu et al., 2021). In this study, it was found that the plants that continued to be cultured after the seed-soaking operation using strain FYN-22 on germinating rice had a significant increase in shoot length, root length, fresh weight, and dry weight, which was more favorable to the growth of rice in the case of direct seeding (Table 7).

The main physiological function of sugars is to provide energy for the animal organism, and they are often added to feed as a cheap source of energy during animal breeding. Fish, especially carnivorous fish, are less able to use sugars compared to terrestrial animals, and some even have certain physiological characteristics innately characteristic of diabetics (Wilson and Poe, 1987). It has been shown that common carp has much higher insulin levels than terrestrial mammals, even in the fasted state (Dixon and Hilton, 1981); in glucose tolerance tests, the vast majority of fish showed persistent symptoms of hyperglycemia after glucose infusion (Hertz et al., 1989). Generally speaking, enteroglucagon has the function of promoting insulin secretion and inhibiting glucagon secretion, thus achieving hypoglycemic effects, but enteroglucagon is susceptible to degradation by dipeptidyl peptidase-IV (DDP-IV) and is inactive (Moon, 2001). Strains with DDP-IV inhibitors inhibit dipeptidyl peptidases and prolong the half-life of the enteric insulin glucagon-like peptide (GLP-1) and glucagon-releasing peptide (GIP), thereby indirectly lowering blood glucose (Matuci and Giampietro, 2009). Therefore, in this study, strains with DDP-IV-producing inhibitors were screened from the RF system with up to 58.73% inhibition (Table 2), and the acid and bile salt tolerance of the strains was measured, indicating that the strains have a higher probability of colonizing the fish gut and, thus, improving the hyperglycemic condition of the fish.

RF acts as a small ecosystem, with microorganisms widely distributed in the fish, water, soil, and plants. Microorganisms attached to water bodies, weeds, and phytoplankton can colonize the gut of common carp through their feeding activities and have an impact on the fish (Zhang et al., 2022). The microorganisms in the fish gut also spread into the water and soil with the fish excretion, while some strains of bacteria can colonize the water body and, thus, improve the water environment. Some studies have shown that the pesticides and fertilizers used in rice cultivation seriously harm the water environment, leaving large amounts of harmful substances in the ecosystem, as well as inducing eutrophication, which can be eliminated to some extent through microbial metabolism (Kong et al., 2022). On the other hand, some strains also settle into the soil, and these microorganisms undertake several important ecosystem functions including soil carbon and nitrogen cycling, helping to alleviate problems such as reduced soil fertility and soil consolidation caused by rice cultivation (Daniel et al., 2022). The microorganisms that settle into the soil not only improve the soil environment, but a proportion of the bacteria also act as plant growth-promoting rhizobacteria (PGPR) to contribute directly or indirectly to the growth and health effects of rice (Bhattacharyya and Jha, 2012; Hacquard et al., 2015). Microorganisms, therefore, play an important role in RF, directly affecting the quality of aquatic products, the water quality soil environment, and the regulation of the growth of rice plants (Kim et al., 2008). In this experiment, targeted screening of strains with DDP-IV-inhibitory ability, IAA activity, and resistance to stress in the RF system could, to some extent, improve hyperglycemia in common carp in the RF system and promote germination of rice seedlings.

## Conclusion

In this study, strain FYN-22 with strong DDP-IV inhibition and IAA production capacity was screened from the intestinal contents of common carp, initially identified as *Bacillus licheniformis*, and verified for its resistance. OFAT experiments were used to screen the types of carbon, nitrogen, and inorganic salts for the FYN-22 fermentation medium and to determine the corresponding ranges. Individual effects and interactions among the four factors screened using OFAT experiments for soluble starch (carbon source), yeast (organic nitrogen source), NH_4_Cl (inorganic nitrogen source), and FeCl_3_ (inorganic salt) were determined using RSM. The spore concentration of *Bacillus licheniformis* FYN-22 at a soluble starch concentration of 10.961 g/l, yeast concentration of 2.366 g/l, NH_4_Cl concentration of 1.881 g/l, and FeCl_3_ concentration of 0.850 g/l reached 1.913 × 10^9^ CFU/ml, which was 2.575-fold greater than the value before optimization. The optimized fermentation broth was used for the immersion culture of rice seeds, and it was found that strain FYN-22 significantly increased the shoot length, root length, fresh weight, and dry weight of rice seedlings, indicating that strain FYN-22 has a good growth-promoting effect. In the future, research will focus on the interaction between the FYN-22 strain and common carp, with practical extension in RF ecosystems and large-scale industrial production.

## Data availability statement

The datasets presented in this study can be found in online repositories. The names of the repository/repositories and accession number(s) can be found at: https://www.ncbi.nlm.nih.gov/genbank/, SUB12101904 FYN-22 OP535852.

## Ethics statement

The animal study was reviewed and approved by the local ethics committee and followed by the European Directive 2010/63/EU for animal experiments.

## Author contributions

BX and HZ designed the experiments. BX, HZ, LL, BZ, YX, YZ, ZS, and SX performed the experiments. BX analyzed the data and prepared the manuscript. SX and YH reviewed the manuscript.

## Funding

This research was funded by the Natural Science Foundation of Heilongjiang Province (C2017013); China Postdoctoral Science Foundation (2017 M6113470).

## Conflict of interest

HZ was employed by China Animal Husbandry Industry Co., Ltd., Beijing, China.

The authors declare that the research was conducted in the absence of any commercial or financial relationships that could be construed as a potential conflict of interest.

## Publisher’s note

All claims expressed in this article are solely those of the authors and do not necessarily represent those of their affiliated organizations, or those of the publisher, the editors and the reviewers. Any product that may be evaluated in this article, or claim that may be made by its manufacturer, is not guaranteed or endorsed by the publisher.
