# Supplementary figures and images for: Screening and fermentation medium optimization of a strain favorable to Rice–fish Coculture
Source: Front Microbiol. 2022 Nov 10;13:1054797. doi: 10.3389/fmicb.2022.1054797 (PMC9802155; doi:10.3389/fmicb.2022.1054797)

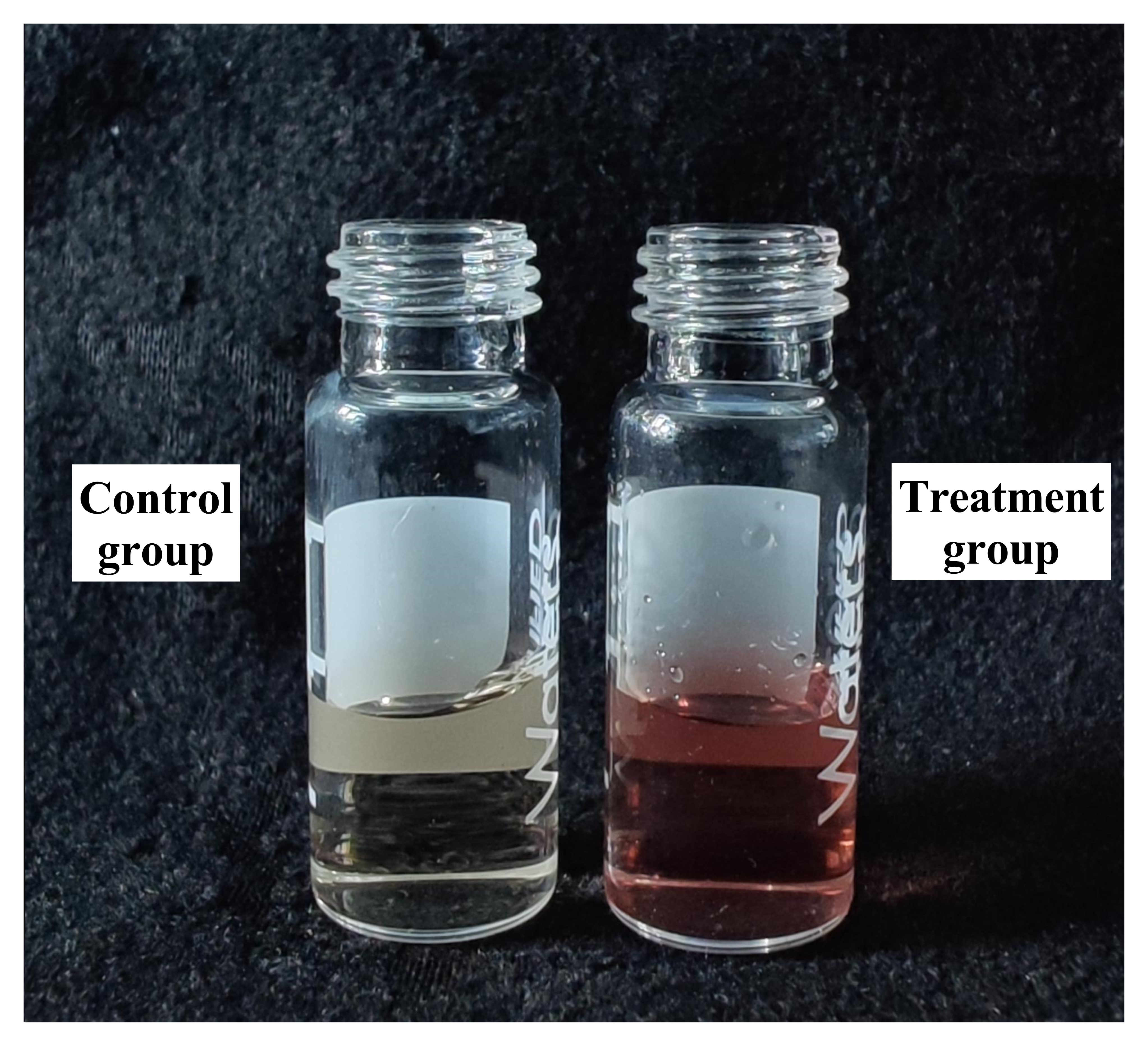

Supplement: Supplementary file 1 [file Image_1.TIF]

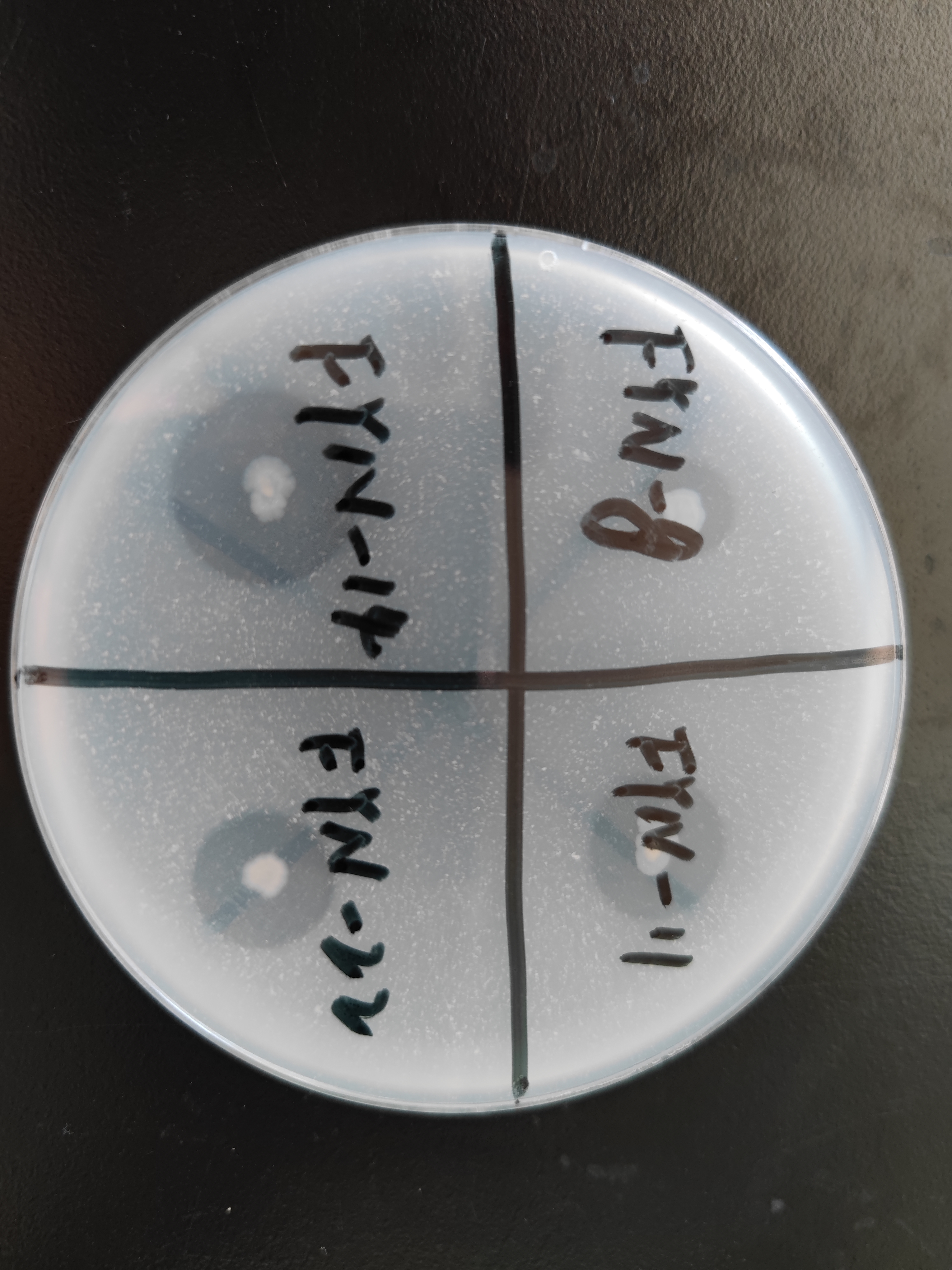

Supplement: Supplementary file 2 [file Image_2.JPEG]
