# Supplementary material for: Screening and fermentation medium optimization of a strain favorable to Rice–fish Coculture
Source: Front Microbiol. 2022 Nov 10;13:1054797. doi: 10.3389/fmicb.2022.1054797 (PMC9802155; doi:10.3389/fmicb.2022.1054797)
Supplement: Supplementary file 3 [file Data_Sheet_1.docx]

Supplementary Material

## 1 Supplementary Figures


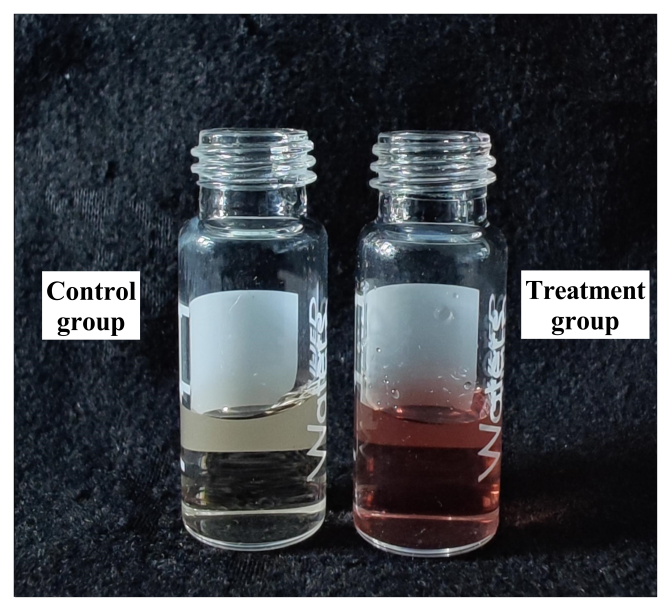


Figure S1. FYN-14 strain DDP-IV-inhibitory capacity.


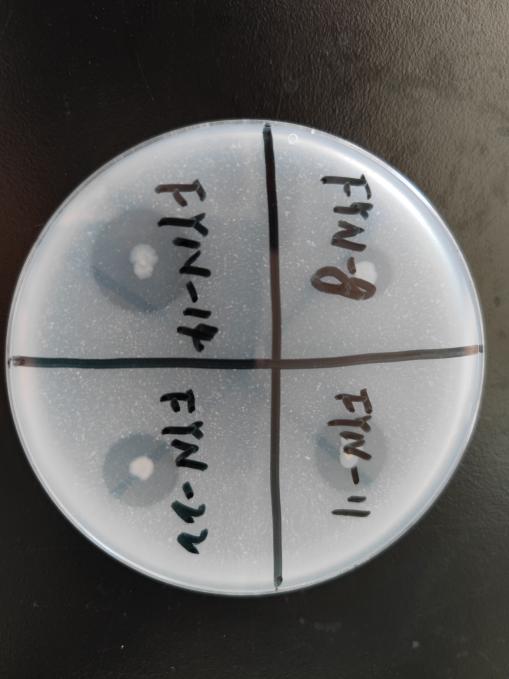


Figure S2. FLN-37 IAA growth hormone-producing capacity.
